# Svx Peptidases of Phytopathogenic Pectolytic Bacteria: Structural, Catalytic and Phytoimmune Properties

**DOI:** 10.3390/ijms25020756

**Published:** 2024-01-07

**Authors:** Natalia Tendiuk, Anastasiya Diakonova, Olga Petrova, Timur Mukhametzyanov, Olga Makshakova, Vladimir Gorshkov

**Affiliations:** 1 Kazan Institute of Biochemistry and Biophysics, FRC Kazan Scientific Center of RAS, 420111 Kazan, Russia; natasha.tendjuk@rambler.ru (N.T.); diakonova27@mail.ru (A.D.); poe60@mail.ru (O.P.); olga.makshakova@kibb.knc.ru (O.M.); 2 Department of Physical Chemistry, Kazan Federal University, 420008 Kazan, Russia; timur.mukhametzyanov@kpfu.ru; 3 Institute of Fundamental Medicine and Biology, Kazan Federal University, 420008 Kazan, Russia

**Keywords:** gluzincin extracellular metallopeptidases, *Pectobacterium*, phytoimmunosuppressors, plant-pathogen interactions, plant susceptible responses, protein structure, Svx proteins, virulence factors

## Abstract

Svx proteins are virulence factors secreted by phytopathogenic bacteria of the *Pectobacterium* genus into the host plant cell wall. Svx-encoding genes are present in almost all species of the soft rot Pectobacteriaceae (*Pectobacterium* and *Dickeya* genera). The Svx of *P. atrosepticum* (*Pba*) has been shown to be a gluzincin metallopeptidase that presumably targets plant extensins, proteins that contribute to plant cell wall rigidity and participate in cell signaling. However, the particular “output” of the *Pba* Svx action in terms of plant-pathogen interactions and plant immune responses remained unknown. The Svx proteins are largely unexplored in *Dickeya* species, even though some of them have genes encoding two Svx homologs. Therefore, our study aims to compare the structural and catalytic properties of the Svx proteins of *Pba* and *D. solani* (*Dso*) and to test the phytoimmune properties of these proteins. Two assayed *Dso* Svx proteins, similar to *Pba* Svx, were gluzincin metallopeptidases with conservative tertiary structures. The two domains of the Svx proteins form electronegative clefts where the active centers of the peptidase domains are located. All three assayed Svx proteins possessed phytoimmunosuppressory properties and induced ethylene-mediated plant susceptible responses that play a decisive role in *Pba*-caused disease.

## 1. Introduction

Phytopathogenic pectolytic bacteria from the soft rot Pectobacteriaceae (SRP), species of the *Pectobacterium* and *Dickeya* genera, cause severe plant diseases worldwide [1]. These bacteria produce a large arsenal of plant cell wall-degrading enzymes (PCWDE) that turn plant tissues into amorphous masses [2]. Due to this, *Pectobacterium* and *Dickeya* are routinely considered “brute force pathogens” [2]. Nevertheless, “stealth behavior” constitutes a large part of the strategy of interaction of these bacteria with plants [3,4,5]. In particular, SRP bacteria are able to interact asymptotically with host plants and weed plants for a long time without applying their brute force potential [6,7,8,9,10,11].

In addition to PCWDEs, SRP bacteria produce other virulence factors, some of which are considered to be more typical of “stealthy” phytopathogens. Without these “other” virulence factors, the pathogenic potential of the SRP bacteria is not manifested to a full extent or even at all, even if PCWDEs are produced to a full level. Type three secretion system [12,13], type five secretion system [14], type six secretion system [15,16], coronofacic acid [17,18,19], siderophores [20,21,22,23] and exopolysaccharides [24] are among these virulence factors.

Some virulence factors of SRP, although identified, remain “black horses” of plant-pathogen interactions because of their unknown particular functions. The Svx protein of *Pectobacterium atrosepticum* (*Pba*) has been shown to be a virulence factor in this species since the knockout of the *svx* gene led to a reduction in *Pba* virulence [25]. The *Pba* Svx protein has also been shown to be secreted via the type two secretion system into the host plant cell wall (PCW), and its production has been demonstrated to be controlled by quorum sensing [15,25]. However, the particular functions that the *Pba* Svx protein implements for providing full virulence remained totally unknown prior to our recent study showing that this protein is a gluzincin metallopeptidase with a zinc-binding active site HEXXHX(22)E and an additional acyltransferase-like domain [26]. Using in silico analyses, we also predicted that *Pba* Svx is a glycopeptidase that is able to digest α-glycosylated proteins that are represented in the PCW with the extensins [26]—PCW proteins that contribute to PCW rigidity and participate in cell signaling [27,28,29]. However, the particular “output” of the *Pba* Svx action in terms of plant-pathogen interactions and plant immune responses remains unexplored.

The *svx*-like genes appeared to be present not only in *Pectobacterium* species but also in a range of phytopathogenic and non-phytobathogenic α-, Δ- and γ-proteobacteria [26]. In non-phytobathogenic bacteria, Svx-like proteins have not been investigated at all. Among phytopathogenic bacteria, the Svx-like protein (AvrXca) was first described in *Xanthomonas campestris* pv. *raphani* as an avirulence factor that can elicit plant immune responses [30]. *Svx*-like genes have been found in many (but not all) *Xanthomonas* species [26]. In turn, within SRP genomes, *svx*-like genes were revealed in all species of both *Pectobacterium* and *Dickeya* genera (except *D. poaceiphila*), indicating that Svx-like proteins play an important role in these bacteria [26]. Interestingly, in some *Dickeya* species (*D. solani*, *D. dadantii*, *D. chrysanthemi* and *D. lacustris*), genes for simultaneously two Svx-like homologs were found, whereas in *Pectobacterium* species, this gene is always present in a single copy [26]. Nevertheless, in *Dickeya* species, these proteins have not been characterized, except that their secretion via the type two secretion system has been demonstrated [31].

Therefore, our study aimed to compare the structural and catalytic properties of the Svx-like proteins of two SRP species (*Pba* and *D. solani* (*Dso*)) and to test the phytoimmune properties of these proteins.

## 2. Results

### 2.1. Comparative Analysis of the Structure of the Svx-like Proteins of Pba and Dso

The pairwise alignment of the amino acid sequences of *Pba* Svx (WP_011092533.1) with two *Dso* Svx-like proteins (WP_022634244.1 (further referred to as *Dso* Svx44) and WP_022634245.1 (further referred to as *Dso* Svx45)) using PSI-protein BLAST revealed that the *Dso* Svx45 protein is more similar to *Pba* Svx (69.03% of sequence identity) than *Dso* Svx44 (55.63% of sequence identity). All three proteins have N-terminal Sec/SPI-type signal peptides (determined by the SignalP 6.0 server). The secondary structure of *Pba* Svx has been determined in our previous study using both in silico modeling and CD-spectroscopy [26]. The secondary structures of *Dso* Svx44 and *Dso* Svx45 were similar to that of the *Pba* Svx (Figure S1).

**Figure 1:**
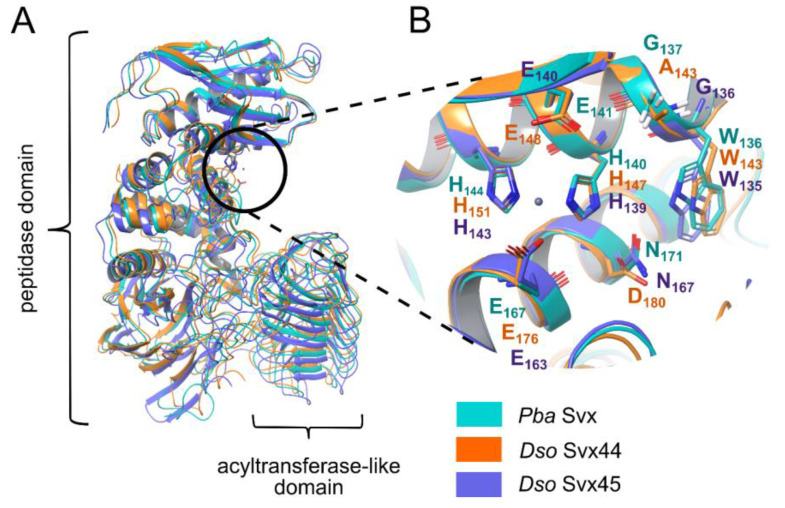
Comparison of the tertiary structure models of the Svx proteins of *Pectobacterium atrosepticum* (*Pba* Svx) and *Dickeya solani* (*Dso* Svx44 and *Dso* Svx45) (**A**) and the active sites of their peptidase domains (**B**). Models were built using the AlphaFold 2 server. Root–mean square deviation values (RMSD) for three comparisons: *Pba* Svx vs. *Dso* Svx44, *Pba* Svx vs. *Dso* Svx45 and *Dso* Svx44 vs. *Dso* Svx45 were 2.92 A, 2.33 A and 2.463 A, respectively. The zinc ion is indicated by a violet sphere.

All three proteins had similar tertiary structures with two functional domains: metallopeptidase and acyltransferase-like (Figure 1A). Similar to *Pba* Svx, the metallopeptidase active sites of *Dso* Svx44 and *Dso* Svx45 are formed by the zinc-binding motif HEXXHX(8,28)E (Figure 1B). The amino acid residues predicted previously to constitute the *Pba* Svx carbohydrate binding site [26] were similar in *Pba* Svx and *Dso* Svx45, whereas in *Dso* Svx44, amino acid substitutions N171D and G137A (the positions of the amino acid residues correspond to the *Pba* Svx) were revealed in the predicted carbohydrate binding site (Figure 1B).

**Figure 2:**
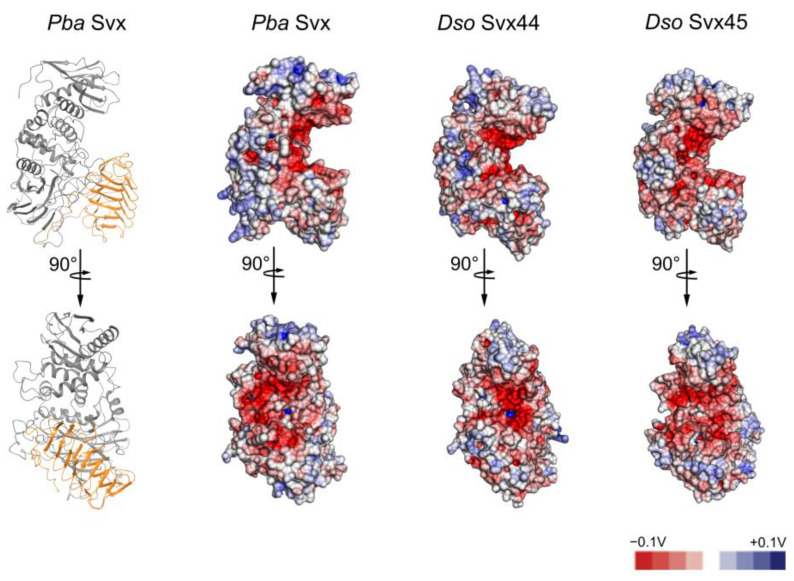
Electrostatic surface maps of the Svx proteins of *Pectobacterium atrosepticum* (*Pba* Svx) and *Dickeya solani* (*Dso* Svx44 and *Dso* Svx45). All surface maps are demonstrated at the same angle as the ribbon structure of the *Pba* Svx protein on the left, where the peptidase domain is indicated in gray and the acyltransferase-like domain is indicated in orange. Red-to-blue colors on electrostatic surfaces correspond to negative-to-positive electrostatic potentials.

The metallopeptidase and acyltransferase-like functional domains in all three proteins interact with each other; herewith, interdomain interactions are stabilized via nonpolar interactions mostly provided by the ILE, LEU, TYR and PRO residues (Table S1). The predicted protein structures (including the interdomain interactions) were verified by molecular dynamics simulation; all protein models were stable in the course of molecular dynamics trajectories (Figure S2). The interactions of the metallopeptidase and acyltransferase-like domains yielded a large electronegative cleft (Figure 2), which accommodated the active site of the peptidase domain.

### 2.2. Catalytic Properties of Dso Svx-like Proteins

**Figure 3:**
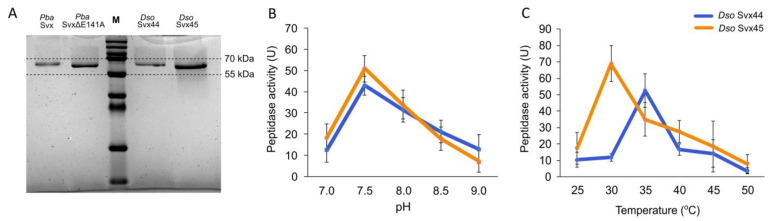
Purified recombinant Svx proteins of *Dickeya solani* (*Dso* Svx44 and *Dso* Svx45) (**A**) and their peptidase activities at different pH values (**B**) and different temperatures (**C**). The pH optimum for the enzymatic activities of both proteins was determined in 100 mM Tris-HCl buffer at 37 °C. The optimal temperature for both proteins was determined in 100 mM Tris-HCl buffer, pH 7.5. One unit of peptidase activity was defined as the amount of enzyme required to produce an absorbance change of 0.1 per min per 1 mg of the corresponding protein. *Pba* Svx in (**A**)—the purified recombinant Svx protein of *Pectobacterium atrosepticum*. *Pba* Svx ΔE141A in (**A**)—the purified recombinant mutant Svx protein of *Pectobacterium atrosepticum* with an amino acid substitution in the active center of the peptidase domain. The values presented are the means ± SD of five replicates.

To verify whether *Dso* Svx44 and *Dso* Svx45, similar to *Pba* Svx [26], possess peptidase activity, recombinant *Dso* Svx44 and *Dso* Svx45 proteins were obtained (Figure 3A). The enzymatic assay with azocasein as a substrate proved that both proteins possessed peptidase activity. Both proteins displayed maximum peptidase activity at pH 7.5 (Figure 3B), similar to *Pba* Svx [26]. *Dso* Svx44 and *Dso* Svx45 peptidase activity was most pronounced at 35 °C and 30 °C, respectively (Figure 3C), whereas *Pba* Svx displayed maximum peptidase activity at 40 °C [26].

**Figure 4:**
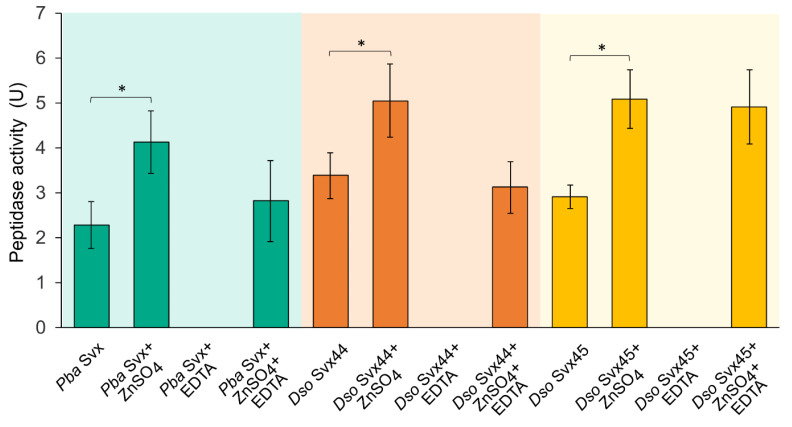
Peptidase activities of the Svx proteins of *Pectobacterium atrosepticum* (*Pba* Svx) and *Dickeya solani* (*Dso* Svx44 and *Dso* Svx45). The activities were measured in the absence (*Pba* Svx, *Dso* Svx44, *Dso* Svx45) or presence of 1 mM ZnSO_4_ (*Pba* Svx+ZnSO_4_; *Dso* Svx44+ZnSO_4_; *Dso* Svx45+ZnSO_4_) or 1 mM EDTA (*Pba* Svx+EDTA; *Dso* Svx44+EDTA; *Dso* Svx45+EDTA) or both 1 mM EDTA and 1 mM ZnSO_4_ (*Pba* Svx+ZnSO_4_+EDTA; *Dso* Svx44+ZnSO_4_+EDTA; *Dso* Svx45+ZnSO_4_+EDTA). For all three proteins, peptidase activity was measured in 100 mM Tris-HCl buffer pH, 7.5. The activities of *Pba* Svx, *Dso* Svx44 and *Dso* Svx45 were assayed at 40 °C, 35 °C and 30 °C, respectively (at the temperature of maximum activity of each protein). One unit of peptidase activity was defined as the amount of enzyme required to produce an absorbance change of 0.1 per min per 1 nmol of the corresponding protein. The presented values are the means ± SD of five replicates. Asterisks (*) show a significant difference (Mann–Whitney two-sided test, *p* < 0.05) between the variants designated by brackets.

To compare the peptidase activities of the three assayed Svx proteins, we performed the enzymatic assay under conditions at which each protein displayed maximum peptidase activity: in 100 mM Tris-HCl buffer (pH 7.5) for all three proteins and at 40 °C, 35 °C and 30 °C for *Pba* Svx, *Dso* Svx44 and *Dso* Svx45, respectively. All three proteins had similar levels of peptidase activity (Figure 4). For all three assayed proteins, peptidase activity increased after the addition of 1 mM ZnSO_4_ (65–75%). The addition of 1 mM EDTA completely inhibited the peptidase activity of all three proteins, while in the presence of both 1 mM ZnSO_4_ and 1 mM EDTA, the peptidase activity was pronounced. Thus, our results proved that similar to *Pba* Svx, *Dso* Svx44 and *Dso* Svx45 are gluzincin metallopeptidases.

### 2.3. Phytoimmune Properties of Svx Proteins of Pba and Dso

#### 2.3.1. Effect of the Svx Proteins on Hydrogen Peroxide Levels in Plant Tissues

**Figure 5:**
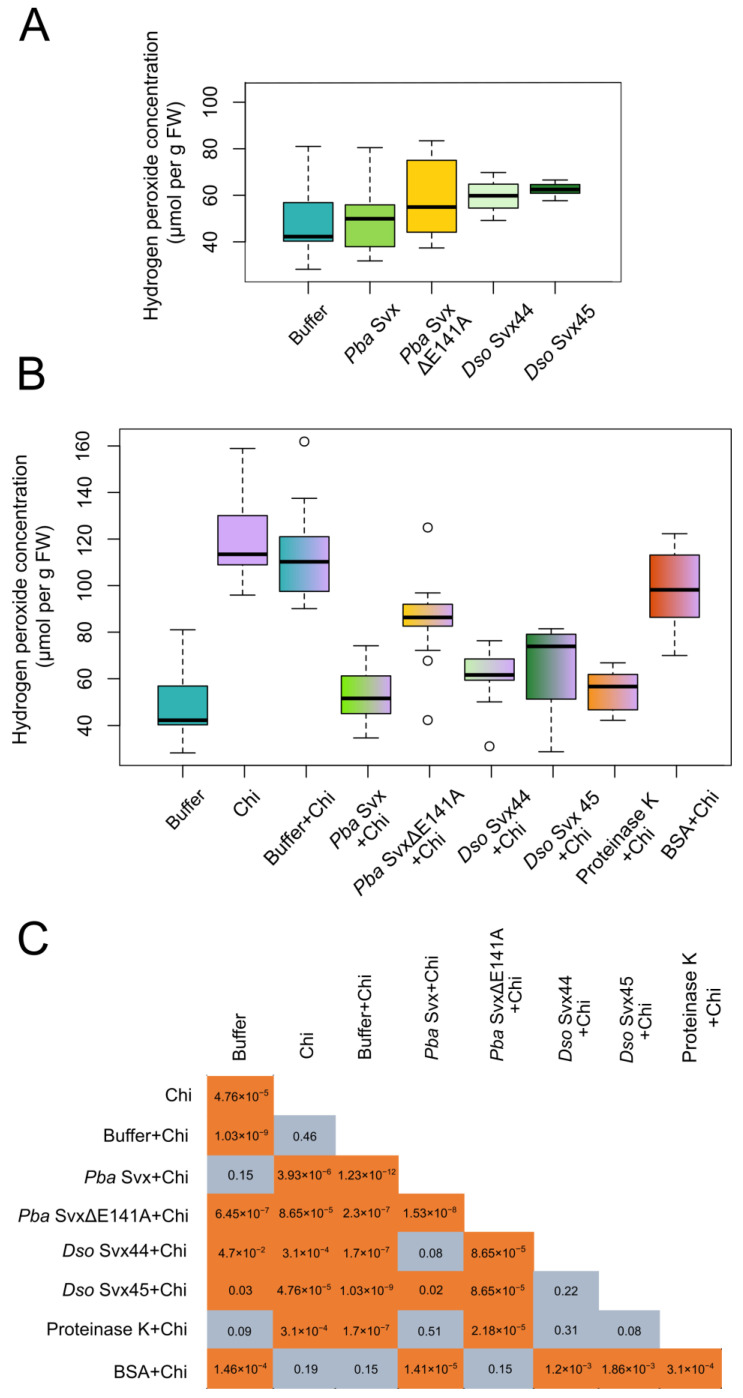
Effect of the Svx proteins of *Pectobacterium atrosepticum* (*Pba* Svx) and *Dickeya solani* (*Dso* Svx44 and *Dso* Svx45) on the level of hydrogen peroxide in tobacco leaves. (**A**) The levels of hydrogen peroxide were measured 12 h after infiltration with 10 mM Tris-HCl, pH 7.5, 50 mM NaCl (dark turquoise), 1 µM *Pba* Svx (salad green), 1 µM *Pba* SvxΔE141A (yellow), 1 µM *Dso* Svx44 (pale green), or 1 µM *Dso* Svx45 (dark green). (**B**) Plant leaves were first infiltrated (except for the variant Chi, purple) with buffer (Buffer, dark turquoise and Buffer+Chi, dark turquoise-purple), 1 µM *Pba* Svx (*Pba* Svx+Chi, salad green-purple), 1 µM *Pba* SvxΔE141A (*Pba* SvxΔE141A+Chi, yellow-purple), 1 µM *Dso* Svx44 (*Dso* Svx44+Chi, pale green-purple), 1 µM *Dso* Svx45 (*Dso* Svx45+Chi, dark green-purple), 1 µM proteinase K (proteinase K+Chi, orange-purple), or 1 µM bovine serum albumin (BSA+Chi, red-purple), and 12 h later, the same leaf areas of all variants (except for the variant Buffer) were infiltrated with 1 µM chitooctaose. The levels of hydrogen peroxide were measured six hours after infiltration with chitooctaose. (**C**) FDR values obtained by statistical analysis (Mann–Whitney test with Benjamini—Hochberg adjustment, FDR < 0.05) of differences in the level of hydrogen peroxide between experimental groups presented in (**B**) (orange background–significant difference; gray background–insignificant difference). No significant differences in the level of hydrogen peroxide were observed between the experimental groups presented in (**A**). All experimental variants were analyzed in at least five biological replicates. FW—fresh weight.

To check whether *Pba* Svx, *Dso* Svx44 and *Dso* Svx45 possessed phytoimmunomodulatory (elicitor) properties, the protein preparations (1 µM) were infiltrated into tobacco leaves, and the hydrogen peroxide level was measured 12 h after infiltration. In addition, we analyzed the effect of a mutant *Pba* Svx protein (*Pba* Svx ΔE141A) with an amino acid substitution in the active center of the peptidase domain and reduced peptidase activity [26]. None of the four assayed proteins induced the accumulation of hydrogen peroxide in the infiltrated plant leaves (Figure 5A).

To analyze possible phytoimmunosuppressory properties, tobacco leaves were non-pretreated or pretreated by infiltration with buffer (control) or 1 µM of assayed proteins (*Pba* Svx, *Dso* Svx44, *Dso* Svx45 and *Pba* Svx ΔE141A), and 12 h later, the same leaf areas were infiltrated with 1 µM of chitooctaose, one of the well-known phytoimmunomodulators [32].

Infiltration with chitooctaose led to an increase in the level of hydrogen peroxide in non-pretreated or buffer-pretreated leaves (Figure 5B,C). In turn, pretreatment with *Pba* Svx completely abolished the chitooctaose-induced accumulation of hydrogen peroxide, whereas pretreatment with *Dso* Svx44 or *Dso* Svx45 significantly reduced the chitooctaose-induced accumulation of hydrogen peroxide (compared to a variant pretreated with buffer) (Figure 5B,C). Pretreatment with the mutant protein *Pba* Svx ΔE141A also reduced chitooctaose-induced accumulation of hydrogen peroxide (compared to a variant pretreated with buffer), but the *Pba* Svx ΔE141A phytoimmunosuppressory effect was significantly less pronounced than that of the wild type *Pba* Svx protein (Figure 5B,C).

As additional controls, we also pretreated plant leaves with 1 µM of bovine serum albumin (BSA) or 1 µM proteinase K. Pretreatment with BSA did not reduce the chitooctaose-induced accumulation of hydrogen peroxide, whereas preinfiltration with proteinase K completely abolished the chitooctaose-induced accumulation of hydrogen peroxide (Figure 5B,C).

#### 2.3.2. Effect of Svx Proteins on the Expression of Infection-Related Genes

**Figure 6:**
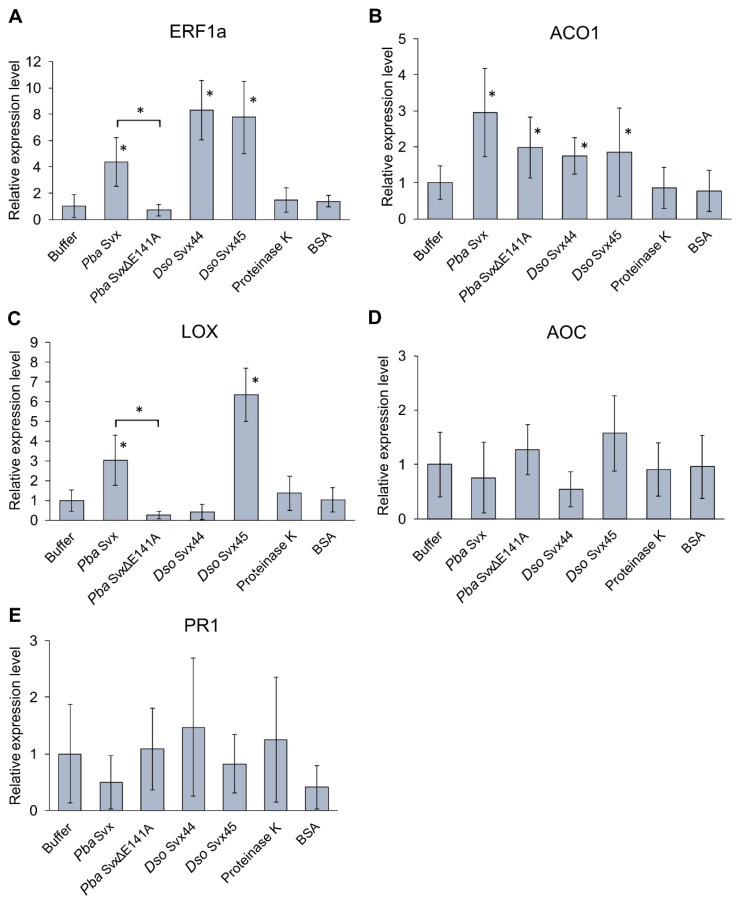
Effect of the Svx proteins of *Pectobacterium atrosepticum* (*Pba* Svx) and *Dickeya solani* (*Dso* Svx44 and *Dso* Svx45) on the expression of tobacco plant genes encoding ethylene-responsive transcription factor 1a (ERF1a) (**A**) and ethylene-biosynthetic enzyme 1-aminocyclopropane-1-carboxylic acid oxidase 1 (ACO1) (**B**) (markers for the ET-mediated hormonal system), JA-biosynthetic enzymes lipoxygenase 2 (LOX2) (**C**) and allene oxide cyclase (AOC) (**D**) (markers for the JA-mediated hormonal system), pathogenesis-related protein 1 (PR1) (**E**) (marker for the SA-mediated hormonal system). Transcript levels of the target genes were determined 12 h after leaf infiltration with 10 mM Tris-HCl, pH 7.5, 50 mM NaCl buffer, (mock-infiltrated control) (Buffer), 1 µM *Pba* Svx, 1 µM mutant *Pba* SvxΔE141A, 1 µM *Dso* Svx44, 1 µM *Dso* Svx45, 1 µM proteinase K, or 1 µM bovine serum albumin (BSA). The expression levels of each gene in the mock-infiltrated control are equated to one. The presented values are the means ± SD of at least eight biological replicates. Asterisks (*) show a significant difference (Mann–Whitney two-sided test with Benjamini–Hochberg adjustment, FDR < 0.05) from the mock-infiltrated control or between the variants designated by brackets.

To investigate whether Svx proteins affect plant responses mediated by ethylene (ET)-, jasmonic acid (JA)- and salicylic acid (SA), phytohormones involved in response to pathogens, the expression levels of marker genes were assessed in tobacco leaves 12 h after protein infiltration. As additional controls, the expression of marker genes was also assessed following infiltration with proteinase K and BSA. The selected marker genes encode ethylene-responsive transcription factor 1a (ERF1a) and ethylene-biosynthetic enzyme 1-aminocyclopropane-1-carboxylic acid oxidase 1 (ACO1) (markers for the ET-mediated hormonal system), JA-biosynthetic enzymes lipoxygenase 2 (LOX2) and allene oxide cyclase (AOC) (markers for the JA-mediated hormonal system), pathogenesis-related protein 1 (PR1) (marker for the SA-mediated hormonal system). Neither proteinase K nor BSA affected the expression level of all five target genes (Figure 6).

All three target proteins (*Pba* Svx, *Dso* Svx44 and *Dso* Svx45) induced the expression of marker genes of the ET-mediated hormonal system, ERF1a and ACO1, especially the gene encoding ERF1a (Figure 6A,B). The mutant *Pba* SvxΔE141A protein also induced the expression of the ACO1 gene but had no effect on the expression of the ERF1a gene. *Pba* Svx and *Dso* Svx45 proteins, but not *Dso* Svx44 and *Pba* SvxΔE141A, induced the expression of the LOX gene (Figure 6C), but none of the assayed proteins affected the expression level of the AOC gene (Figure 6D) as well as the marker for the SA-mediated hormonal system PR1 (Figure 6E).

## 3. Discussion

In the present study, we compared the structures and catalytic properties of the Svx proteins of the species of two genera of the soft rot Pectobacteriaceae, *Pba* and *Dso*, and explored the phytoimmune properties of these proteins. *Pba* (as well as all *Pectobacterium* species) has a single gene encoding the Svx protein, whereas *Dso* (as well as several *Dickeya* species) has two Svx protein-encoding genes. Therefore, three Svx proteins (*Pba* Svx, *Dso* Svx44 and *Dso* Svx45) were analyzed in this study.

*Pba* Svx was first characterized in our recent study, where we have shown that this protein is a zinc-dependent metallopeptidase with an additional acyltransferase-like domain. Besides, *Pba* Svx has been predicted to be a glycopeptidase, which is presumably able to hydrolyze α-glycosylated proteins that are represented in the PCW with the extensins [26].

In the present study, we showed that the Svx proteins of *Dso* (*Dso* Svx44 and *Dso* Svx45) are also zinc-dependent metallopeptidases with similar catalytic properties to those of *Pba* Svx, except that their maximum levels of peptidase activities are manifested at lower temperatures (30–35 °C) than in *Pba* Svx (40 °C). All three proteins have similar tertiary structures with two functional domains (peptidase and acyltransferase-like) and possible carbohydrate-binding residues. Modeling of the tertiary structures showed that two domains of the assayed proteins interact with each other predominantly via hydrophobic interactions, forming large electronegative clefts where the active sites of the peptidase domains are accommodated.

The location of the active sites of peptidase domains in electronegative clefts indicates that proteins with positively charged surfaces formed by arginine, lysine, or histidine residues or positively charged carbohydrate residues are preferable substrates of Svx proteins. Among the extensins (that were predicted in our previous study to be the substrates of the *Pba* Svx protein [26]), those that have lysine-rich positively charged domains are known [33]. The extensin net *in muro* is considered to form positively charged scaffolds that react with negatively charged pectins to create an extensin-pectate coacervate [33]. Lysine residues, which are usually located between the α-glycosylated sites of extensins, can presumably interact with the negatively charged surface of the Svx pocket and hold the α-glycosylated extensin region within the catalytic site of the Svx protein. Therefore, our present study strengthens the hypothesis that some extensins are possible targets of the Svx peptidases.

Besides the extensins, other plant cell wall proteins can have positively charged elements, e.g., proteins N-glycosylated with N-acetylglucosamine and lysine/histidine-rich arabinogalactan proteins [34,35,36,37]. However, the topology of the carbohydrate-binding site of *Pba* Svx restricts its interactions with N-glycosylated proteins and β-glycosylated arabinogalactan proteins [26]. Therefore, α-glycosylated extensins with negatively charged domains remain the most plausible substrates of Svx proteins.

The analysis of the immune properties showed that the Svx proteins of *Pba* and *Dso* did not have phytoimmunomodulatory (elicitor) properties as they did not induce the accumulation of hydrogen peroxide in plants. In contrast, the first-described *svx* gene (*avrXca*) in *Xanthomonas campestris* pv. *raphani* has been shown to encode a protein that induces a strong phytoimmune response [30].

All three assessed proteins possessed phytoimmunosuppressive properties since they reduced the hydrogen peroxide level elicited by chitooctaose. Herein, the mutation in the zinc-binding active site reduced the phytoimmunosuppressive properties of *Pba* Svx (*Pba* SvxΔE141A). This means that peptidase activity is important for the phytoimmunosuppressive properties of this protein. It cannot be ruled out that the observed phytoimmunosuppressive effect of the Svx proteins could be partially related to the non-specific peptidase activity since a similar molar concentration of proteinase K also resulted in the reduction of hydrogen peroxide levels following elicitation with chitooctaose. However, it should be noted that proteinase K possessed more than two orders of magnitude greater peptidase activity than similar molar concentrations of the assayed Svx proteins; and therefore, proteinase K could lead to dramatic destruction of apoplastic proteins, including those involved in the generation of reactive oxygen species. In turn, given the relatively low peptidase activities of the Svx proteins (compared to proteinase K), their high phytoimmunosuppressive effect is likely related to the specificity of their action toward particular proteins involved in the immune response.

A more specific effect of the Svx proteins was observed in the regulation of infection-associated gene expression. All three Svx proteins induced the expression of the ethylene-biosynthetic gene and especially the ethylene-responsive gene ERF1a, and such an effect has not been observed for proteinase K. The mutant protein *Pba* SvxΔE141A provided some induction of the ethylene-biosynthetic gene; however, the expression of the ethylene-responsive gene ERF1a was not affected by SvxΔE141A, indicating that peptidase activity is necessary for Svx proteins to induce ethylene-mediated responses.

Ethylene responses are often considered from the perspective of their defense role during plant-pathogen interactions [38]. However, in many cases, ethylene-mediated reactions reflect plant susceptible responses–host reactions driven by pathogen manipulation in order to either “improve” its ecological niche, or cause disease, or both [39]. In the case of plant-*Pba* interaction, ethylene-mediated responses were definitely demonstrated to promote disease development, being a susceptibility factor [19]. Therefore, our study shows that Svx proteins participate in the induction of ethylene-mediated susceptible responses in plants during their interactions with SRPs.

Other factors of plant susceptibility to *Pba* are responses regulated by one of a variety of products of the lipoxygenase cascade, jasmonates [18,19]. We revealed that *Pba* Svx and *Dso* Svx45 but not *Dso* Svx44 and *Pba* SvxΔE141A induced the expression of the gene encoding lipoxygenase, which is a start enzyme of the lipoxygenase cascade. However, none of the assayed proteins induced the expression of a gene encoding allene oxide cyclase, the enzyme that catalyzes the final step of the synthesis of jasmonic acid. This means that *Pba* Svx and *Dso* Svx45 can presumably affect some reactions of the lipoxygenase cascade that is typical of plant-*Pba* interactions [18]; however, the jasmonic acid-biosynthetic branch of the lipoxygenase cascade is unlikely to be affected by the target proteins.

Whether ethylene and jasmonates promote plant susceptibility or resistance to *Dickeya* species remains debatable and poorly investigated [23]. However, several experimental studies testify in favor of the hypothesis that these two phytohormones contribute more to plant susceptibility to *Dickeya* species than to resistance to these pathogens, at least at some stages of plant-pathogen interactions [23].

SA is well-reported to promote plant resistance to both *Pectobacterium* and *Dickeya* species [19,40,41,42,43]. Neither of the Svx proteins assayed in our study induced the expression of the SA-pathway marker, the PR1 gene. This shows that Svx proteins do not activate the defense pathway, which restricts the propagation of these bacteria *in planta*.

Among the virulence factors of phytopathogenic microorganisms, many proteins with metallopeptidase activity have been described [44,45,46,47,48,49,50,51,52,53,54]. Most of these virulence-related metallopeptidases are considered “instruments” of brute force that contribute to the disintegration of plant tissues, providing non-specific, extensive digestion of host proteins [45,46]. However, some virulence-related metallopeptidases have been reported to be targeted at particular host proteins (e.g., chitinases, extensins, feredoxins), acting presumably in a “stealth-like” manner [47,48,49,50,51]. Some of these metallopeptidases affect host immune responses. For example, Avr-Pita metallopeptidase from *Magnaporthe grisea* and *Magnaporthe oryzae* targets the cytochrome c oxidase assembly protein OsCOX11, a key regulator of mitochondrial reactive oxygen species metabolism [52,53]. Hydrolysis of OsCOX11 by Avr-Pita results in suppression of pathogen-associated molecular pattern (PAMP)-triggered defense responses and an increase in plant susceptibility. Herewith, in the presence of the specific host R protein, Avr-Pita acts as a race-specific elicitor that triggers a hypersensitive response associated with full plant resistance [53]. FgFly1 metallopeptidase from *Fusarium graminearum* interacts with the calmodulin-binding transcription activator, inhibiting the SA-mediated immune response [54].

The Svx peptidases characterized in our study are unlikely to be directly involved in the “brute force” of *Pectobacterium* and *Dickeya* species since these enzymes possess relatively low peptidase activity and we have never observed any signs of plant tissue damage following infiltration of these proteins. However, Svx peptidases are likely to be indirectly involved in “brute force” since they promote host plant responses (ethylene-mediated reactions) that play an important role in plant susceptibility to these pathogens and their aggressive behavior (at least *Pba*) [19].

## 4. Materials and Methods

### 4.1. In Silico Analysis of the Protein Structure 

Pairwise alignment of the amino acid sequences was performed using PSI-protein BLAST [55,56]. The presence of signal peptides was analyzed using the SignalP 6.0 server [57,58]. Multiple alignments were carried out in the MAESTRO 12.1 software [59]. Tertiary structures of the proteins were predicted by the AlphaFold 2 server [60,61] (Figure S3). The electrostatic potential of proteins was calculated using the eF-surf server [62,63].

### 4.2. Molecular Dynamics Simulations

The structures of all three proteins were equilibrated using molecular dynamics (MD). MD simulations were carried out in the Amber18 package using the Amber14SP force-field parameters [64]. Protein molecule was immersed into a water box with periodic boundary conditions. The TIP3P model was used for water molecules. Sodium ions were added to the amount necessary to keep system’s electroneutrality. The integration step of 2 fs was used together with the SHAKE algorithm constraining the bonds involving hydrogen atoms [65]. The Particle Mesh Ewald (PME) method was used for long-ranged electrostatic interactions [66]. The simulations were carried out in the isotherm isobar thermodynamic ensemble at 300 K. The temperature and the pressure were kept constant using a Langevin thermostat with a collision frequency of 2 ps^−1^ and a weak coupling algorithm with a relaxation time of 2 ps, respectively. First, the system was minimized for 5000 steps and then equilibrated. In the production run 700 ns of the trajectory were accumulated.

### 4.3. Gene Cloning and Protein Purification

Since the target genes A4U42_RS02180 (*Dso svx44*) and A4U42_RS02185 (*Dso svx45*) in *Dickeya solani* IPO2222 have similar 5′- and 3′-ends, their separate PCR-amplification (without signal-peptide coding sequences) was performed in two-stage PCR. First, to avoid simultaneous amplification of both target DNA fragments, the primers Svx44_F/Svx44_R and Svx45_F/Svx45_R (Table S2) complementary to the unique DNA regions adjacent to the open reading frames (ORF) of the target genes were used. Then, the obtained PCR products were separately used as templates for PCR with primers Svx44His_F/Svx44His_R and Svx45His_F/Svx44His_R (Table S2) to separately amplify the *svx44* and *svx45* ORFs without sequences encoding signal peptides. Primers Svx44His_R and Svx44His_R at 3′-ends also contained the sequences encoding C-terminal 6His-tags. The aforementioned PCR reactions were performed under the following conditions: 98 °C for 30 s, followed by 25 cycles at 98 °C for 10 s, 68 °C for 15 s and 72 °C for 60 s using Q5 polymerase (NEB, Ipswich, MA, USA). The obtained DNA fragments were ligated with pET51b vectors (Merck KGaA, Darmstadt, Germany).

Cells of *Escherichia coli* strain BL21 (DE3) (Invitrogen, Waltham, MA, USA) were transformed with the resultant pET51b-Svx44 and pET51b-Svx45 plasmids and grown in sterile LB:M9 (1:1) media supplemented with 100 µg/mL ampicillin at 37 °C until the cultures reached a density of ~0.6 at 600 nm. Synthesis of the recombinant proteins was induced by the addition of isopropyl-β-D-1-thiogalactopyranoside (IPTG) (Thermo Fisher Scientific, Waltham, MA, USA) up to 0.5 mM concentration. Cultures were incubated overnight at 18 °C with shaking. Bacterial cells were collected, and cell lysis was performed using the BugBuster Protein Extraction Reagent (Merck KGaA, Darmstadt, Germany), 0.1 mg/mL of lysozyme (Thermo Fisher Scientific, Waltham, MA, USA) and 0.1 mg/mL of DNase I (Bio-Rad, Hercules, CA, USA) at 37 °C for 20 min. Cell lysates were centrifuged (12,000× *g*, 4 °C, 30 min), and the inclusion bodies were washed with buffer (100 mM Tris-HCl, pH 8.0, 150 mM NaCl) and pelleted again (12,000×*g*, 4 °C, 30 min). The pellet was dissolved in a denaturing buffer (100 mM Tris-HCl, 150 mM NaCl, 8 M urea, pH 8.0) and loaded on chromatography columns with HisPur™ Ni-NTA Resin (Thermo Fisher Scientific, Waltham, MA, USA). Non-target proteins were removed from the columns with washing buffer (100 mM Tris-HCl, 150 mM NaCl, 25 mM imidazole, 8 M urea, pH 8.0) and target proteins were eluted with elution buffer (100 mM Tris-HCl, 150 mM NaCl, 250 mM imidazole, 8 M urea, pH 8.0). Then, one volume of the purified recombinant proteins was added dropwise to 20 volumes of the corresponding refolding buffer: for *Dso* Svx44–100 mM Tris-HCl, 150 mM NaCl, 1.25 mM reduced glutathione, 1.25 mM oxidized glutathione, 0.25 M arginine and 1 mM ZnSO_4_; for *Dso* Svx45–100 mM Tris-HCl, 150 mM NaCl, 0.5 mM reduced glutathione, 2 mM oxidized glutathione, 0.5 M arginine and 1 mM ZnSO_4_ at 4 °C for 12 h. The resultant solution was clarified by centrifugation at 10,000× *g* for 30 min. All procedures for obtaining the recombinant proteins were performed at 4 °C. For further assays (enzymatic activity, phytoimmune properties), the buffer in protein preparations was changed to 10 mM Tris-HCl, 50 mM NaCl, pH 7.5 using Zeba spin desalting columns (Thermo Fisher Scientific, Waltham, MA, USA). The recombinant proteins *Pba* Svx and its mutant form *Pba* Svx ΔE141A with an amino acid substitution in the active center of the peptidase domain and reduced peptidase activity were obtained as described in our previous study [26].

The obtained recombinant proteins were analyzed by SDS-PAGE [67] using an acrylamide concentration of 13% and visualized with Coomassie R-250 (Sigma-Aldrich, St. Louis, MO, USA). Protein concentrations were determined using a Qubit fluorimeter (Thermo Fisher Scientific, Waltham, MA, USA).

### 4.4. Peptidase Activity Assays

For the peptidase activity assays, azocasein was used as the substrate (Sigma-Aldrich, St. Louis, MO, USA) [68]. Ten μg of the recombinant proteins (in 100 μL) was incubated (60 min) in 250 μL of 0.5% azocasein in 100 mM Tris-HCl buffer with a pH range of 7.0–9.0 and a temperature range of 25–50 °C. When specified, the reaction mixtures were supplemented with 1 mM ZnSO_4_ and/or 1 mM EDTA. The reactions were stopped by the addition of 100 μL of 10% trichloroacetic acid (Sigma-Aldrich, St. Louis, MO, USA). The sediment was removed by centrifugation (5000× *g*, 10 min, 25 °C). Supernatants (400 μL) were mixed with 1.0 N NaOH (133 μL), and the absorbance at 440 nm was measured using the microplate reader CLARIOstar (BMG Labtech GmbH, Ortenberg, Germany). One unit of peptidase activity was defined as the amount of enzyme required to produce an absorbance change of 0.1 per min per 1 mg or per 1 nmol of the corresponding protein.

### 4.5. Determination of the Hydrogen Peroxide Level

Tobacco plants (*Nicotiana tabacum* Petit Havana SR1) were grown in soil (Borresources, Kerzhenets, Russia) in 50 mL pots for five weeks at 22 °C under a 16/8 light/dark regime. To assess the phytoimmunomodulatory effect of the target proteins, fully expanded plant leaves were infiltrated with the following solutions: (1) 10 mM Tris-HCl, 50 mM NaCl buffer, pH 7.5 (control), (2) 1 µM *Pba* Svx protein, (3) 1 µM mutant *Pba* SvxΔE141A protein, (4) 1 µM *Dso* Svx44 protein, or (5) 1 µM *Dso* Svx45 protein. Twelve hours after infiltration, the hydrogen peroxide levels were measured in the infiltrated parts of the leaves.

To assess the phytoimmunosuppressory effect of the target proteins, plant leaves were infiltrated with: (1) 10 mM Tris-HCl 50 mM NaCl buffer, pH 7.5 (control), (2) non-infiltrated, (3) 1 µM *Pba* Svx protein, (4) 1 µM mutant *Pba* SvxΔE141A protein, (5) 1 µM *Dso* Svx44 protein, (6) 1 µM *Dso* Svx45 protein, (7) proteinase K (Thermo Fisher Scientific, Waltham, MA, USA), or (8) bovine serum albumin (BSA) (BioClot GmbH, Bayern, Aidenbach, Germany), and 12 h later, the infiltrated leaf areas were again infiltrated (except for the control variant) with 1 μM chitooctaose (Biosynth Ltd., Compton, Newbury RG20, UK). Six hours after the infiltration of chitooctaose, the hydrogen peroxide levels were measured in the infiltrated parts of the leaves.

Hydrogen peroxide levels were determined by a method based on the peroxide-mediated oxidation of Fe^2+^ followed by the reaction of Fe^3+^ with xylenol orange (Sigma, St. Louis, MO, USA). Leaves (100 mg) were ground in 1 mL of a cold 50 mM borate buffer (pH 8.4) in mortars. The homogenates were centrifuged (7000× *g*, 10 min), and 100 μL of the supernatants was added to 500 μL of the assay reagent that contained 500 mM ammonium ferrous sulfate, 50 mM H_2_SO_4_, 200 mM xylenol orange and 200 mM sorbitol. The absorbance of the Fe^3+^–xylenol orange complex (A560) was detected after 45 min using a microplate reader CLARIOstar (BMG Labtech GmbH, Ortenberg, Germany). Standard curves were obtained by adding various amounts of hydrogen peroxide to 100 mL of a borate buffer mixed with 500 mL of the assay reagent. Data were normalized and expressed as µmol hydrogen peroxide per gram of fresh weight [32]. The experiments were performed in at least ten biological replicates. The statistical significance of differences was assessed using a Mann–Whitney two-sided test with a Benjamini-Hochberg adjustment, with FDR < 0.05 considered to be statistically significant.

### 4.6. Gene Expression Analysis Using qRT-PCR

Tobacco plants were grown as described above, and fully expanded plant leaves were infiltrated with: (1) 10 mM Tris-HCl 50 mM NaCl buffer, pH 7.5 (control), (2) 1 µM *Pba* Svx protein, (3) 1 µM mutant *Pba* SvxΔE141A protein, (4) 1 µM *Dso* Svx44 protein, (5) 1 µM *Dso* Svx45 protein, (6) proteinase K (Thermo Fisher Scientific, Waltham, MA, USA), or (7) bovine serum albumin (BSA) (BioClot GmbH, Bayern, Aidenbach, Germany). Twelve hours after infiltration, total RNA was extracted from the infiltrated parts of the leaves. Plant material was ground in liquid nitrogen in mortars. The obtained powder was resuspended in 1 mL of ExtractRNA Reagent (Evrogen, Moscow, Russia), and the subsequent procedures were performed according to the manufacturer’s instructions. To eliminate residual genomic DNA, RNA samples were treated with DNAse I using a DNA-free kit (Thermo Fisher Scientific, Waltham, MA, USA). RNA quantity and quality were analyzed using a NanoPhotometer NP80 (Implen, München, Germany) and electrophoresis in a 1% agarose gel, respectively. One microgram of the total RNA was used for cDNA synthesis using RevertAid reverse transcriptase (Thermo Fisher Scientific, Waltham, MA, USA) according to the manufacturer’s instructions. Two µL of fivefold-diluted cDNA was added to the qRT-PCR mixture. qRT-PCR was performed using the EVA-Green-containing master mix (Syntol, Moscow, Russia) according to the manufacturer’s instructions. Primers for target and reference genes (Supplementary Table S2) were designed using Vector NTI Version 9 software (Invitrogen, Waltham, MA, USA) and synthesized by Evrogen (Moscow, Russia).

PCR was performed under the following conditions: 95 °C for 2 min, followed by 45 cycles at 94 °C for 10 s, 60 °C for 15 s and 72 °C for 30 s. After that, melt curve analysis was performed in the temperature range of 60 to 95 °C. The reactions were run, and changes in fluorescence emission were detected using a CFX96 quantitative PCR system (Bio-Rad, Hercules, CA, USA). The amount of fluorescence was plotted as a function of the PCR cycle using CFX Manager Software (Bio-Rad, Hercules, CA, USA). The amplification efficiency (E) for all primers was determined using a dilution series of a pool of cDNAs. Additional controls included omission of reverse transcriptase to measure the extent of residual genomic DNA contamination and omission of a template. Plant genes encoding elongation factor 1-α and the β-subunit of ATP synthase were used for normalization of the expression of the target genes. Relative expression levels were determined as the ratios between the quantities of cDNA corresponding to the target and reference genes. Experiments were performed in at least eight biological replicates. The statistical significance of differences was assessed using a Mann–Whitney two-sided test with a Benjamini-Hochberg adjustment, with FDR < 0.05 considered to be statistically significant.

## 5. Conclusions

*Dso* Svx44 and *Dso* Svx45, similar to *Pba* Svx, are gluzincin metallopeptidases with a conservative tertiary structure and similar catalytic properties, indicating that Svx proteins implement similar functions in different SRPs. Two domains of the Svx proteins (peptidase and acyltransferase-like) form electronegative clefts where the active centers of the peptidase domains are located, indicating that preferable substrates of the Svx proteins are likely to be proteins with positively charged surfaces. This fact additionally strengthens our previous hypothesis that the plant extensins are targets of the Svx proteins.

Svx proteins possess phytoimmunosuppressory properties; however, whether these properties are determined by non-specific peptidase activity or specific Svx-mediated hydrolysis of particular proteins requires more in-depth investigation. Svx proteins provide the induction of the ethylene-mediated plant susceptible responses that play a decisive role in disease progression following *Pba* infection. Our results form a basis for searching for specific proteins targeted by Svx proteins and deciphering the entire mechanism of their action during disease development.

## Data Availability

Data are contained within the article and supplementary materials.

## Supplementary Materials

The following supporting information can be downloaded at: https://www.mdpi.com/article/10.3390/ijms25020756/s1.
