# Supplementary material for: Svx Peptidases of Phytopathogenic Pectolytic Bacteria: Structural, Catalytic and Phytoimmune Properties
Source: Int J Mol Sci. 2024 Jan 7;25(2):756. doi: 10.3390/ijms25020756 (PMC10815107; doi:10.3390/ijms25020756)
Supplement: Supplementary file 1 [file ijms-25-00756-s001.zip › ijms-2784705-supplementary.pdf]

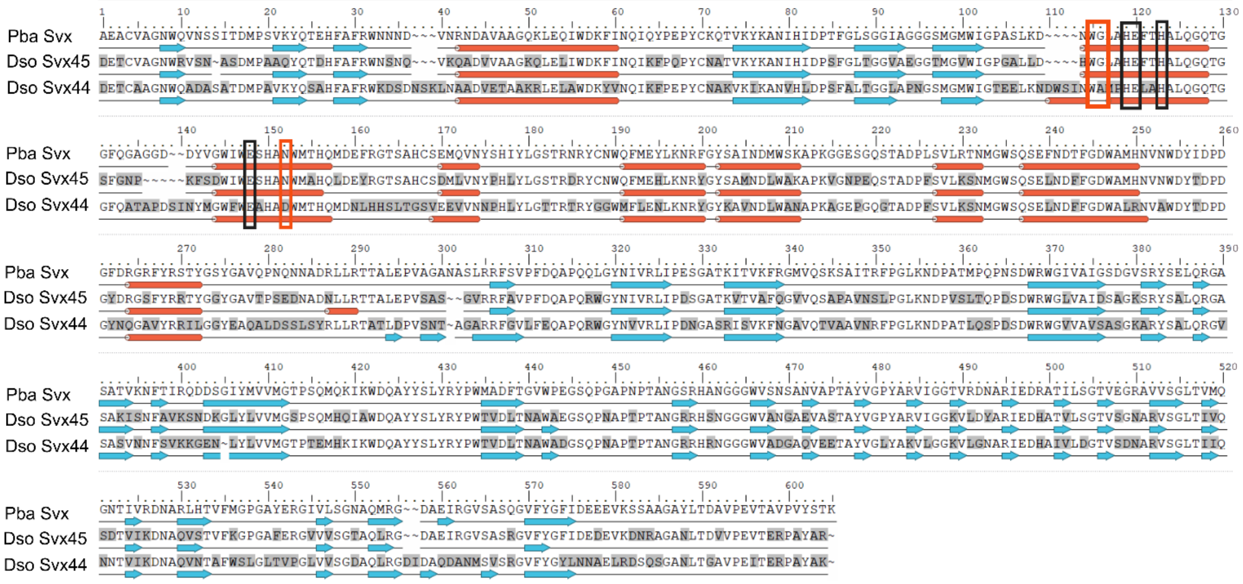

Figure S1. The alignment of amino acid sequences without signal peptides of the *Pectobacterium atrosepticum* (*Pba*) Svx protein (*Pba* Svx) and *Dickeya solani* (*Dso*) Svx-like proteins (*Dso* Svx44 and *Dso* Svx45). The secondary structures predicted by AlphaFold 2 are indicated by orange horizontal cylinders (alpha helices) and blue arrows (beta-strands). Zinc-binding metallopeptidase active sites and predicted carbohydrate-binding residues are indicated by black and bright-orange frames, respectively. Amino acid residues of the *Dso* Svx44 and *Dso* Svx45 that do not correspond to the amino acid residues of the *Pba* Svx are colored gray.

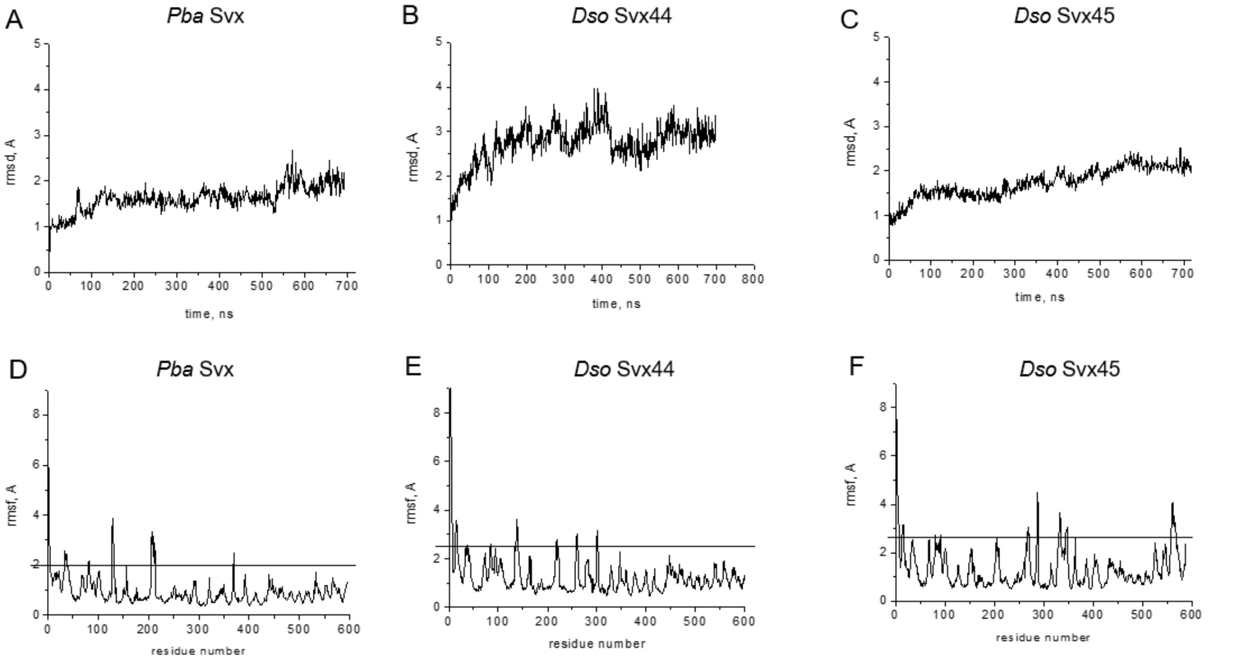

Figure S2. Root-mean square deviation (A, B, C) and root-mean square fluctuation (D, E, F) evolution over time in the course of molecular dynamics trajectories for AlphaFold 2 models of the Svx proteins of *Pectobacterium atrosepticum* (*Pba* Svx) and *Dickeya solani* (*Dso* Svx44 and *Dso* Svx45). The horizontal lines indicate a double value of the trajectory averaged rmsf value.

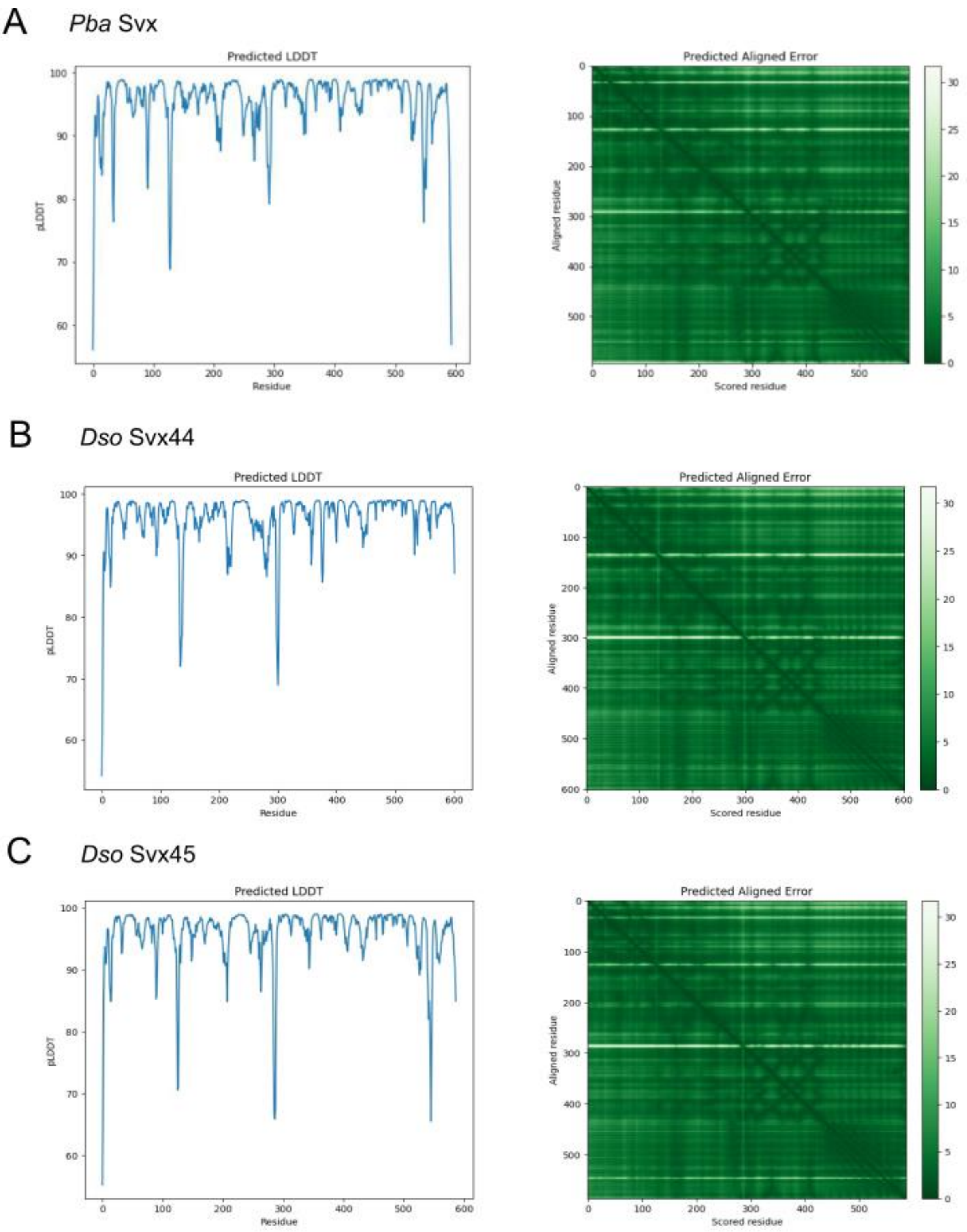

Figure S3. The results of the predicted local-distance difference test for each residue position of the models (built by AlphaFold 2) of the Svx proteins of *Pectobacterium atrosepticum* (*Pba* Svx) and *Dickeya solani* (*Dso* Svx44 and *Dso* Svx45). Data for *Pba* Svx were previously published in supplementary materials [Tendiuk *et al*., 2022]. The model confidence is high, with LDDT above 70% for the majority of the residues. The regions of low confidence (slightly below 70%) are located on the loops.

Table S1. Residues forming contacts between the metallopeptidase and acyltransferase-like domains of the Svx proteins of *Pectobacterium atrosepticum* (*Pba* Svx) and *Dickeya solani* (*Dso* Svx44 and *Dso* Svx45). Shortest distances from a residue to the neighboring domain over time of trajectories are given with cut-off 0.3 nm.

	Residue	Distance (nm)	± Standard Deviation (nm)
Pba Svx			
Metallopeptidase domain	ARG203	0.180	0.010
	ASP331	0.191	0.015
	HIS196	0.192	0.013
	LYS374	0.202	0.058
	GLN301	0.225	0.039
	PRO299	0.228	0.015
	LEU308	0.229	0.015
	GLN332	0.232	0.017
	TYR445	0.232	0.016
	LEU307	0.236	0.018
	GLN298	0.241	0.074
	ALA304	0.241	0.022
	TYR194	0.243	0.018
	ILE197	0.243	0.019
	GLN466	0.250	0.030
	PRO329	0.265	0.029
	PRO315	0.268	0.032
	ALA312	0.273	0.052
	TYR446	0.273	0.018
	ASN300	0.284	0.044
	PRO467	0.296	0.030
acyltransferase-like domains	SER533	0.224	0.014
	VAL553	0.234	0.030
	LEU535	0.235	0.019
	THR552	0.235	0.015
	PRO557	0.237	0.023
	TYR589	0.249	0.019
	VAL537	0.249	0.092
	GLY499	0.278	0.062
	PHE591	0.283	0.081
	ASP575	0.284	0.101
	THR520	0.288	0.102
	ARG503	0.289	0.113
	PHE554	0.297	0.060
Dso Svx44			
Metallopeptidase domain	LYS382	0.193	0.040
	ARG212	0.196	0.025
	HIS205	0.204	0.026
	SER313	0.218	0.041
	GLU339	0.221	0.031
	LEU337	0.234	0.018
	LEU317	0.236	0.018
	TYR314	0.237	0.023

	LEU316	0.239	0.029
	TYR452	0.241	0.017
	ASN203	0.243	0.020
	THR321	0.249	0.027
	GLU199	0.251	0.042
	LEU206	0.253	0.032
	ASP323	0.258	0.095
	LEU308	0.267	0.107
	TYR453	0.269	0.019
	PRO324	0.280	0.054
	SER310	0.299	0.120
acyltransferase-like domain	ALA560	0.202	0.02
	ASP582	0.204	0.031
	HIS525	0.221	0.037
	LEU542	0.224	0.014
	TRP562	0.234	0.030
	ILE544	0.235	0.019
	PHE561	0.235	0.015
	LEU566	0.237	0.023
	GLN546	0.249	0.092
	GLY541	0.268	0.063
	GLY581	0.269	0.025
	TYR508	0.278	0.062
	TYR600	0.283	0.081
	ASP584	0.284	0.101
	LEU529	0.287	0.102
	LEU512	0.289	0.113
	SER563	0.297	0.059
Dso Svx45			
Metallopeptidase domain	ARG199	0.213	0.055
	HIS192	0.224	0.027
	LEU304	0.226	0.015
	ALA300	0.227	0.016
	ASP325	0.230	0.023
	LEU193	0.233	0.018
	TYR190	0.242	0.018
	LEU303	0.243	0.029
	GLU310	0.244	0.086
	PRO323	0.245	0.030
	GLN326	0.247	0.030
	ASP301	0.248	0.059
	PRO295	0.252	0.044
	TYR439	0.259	0.028
	MET186	0.273	0.073
	PRO311	0.274	0.046
	GLU297	0.284	0.098
	TYR440	0.285	0.045
	ALA308	0.288	0.068
	LYS368	0.295	0.161
acyltransferase-like	VAL547	0.216	0.018

domain	HIS512	0.217	0.028
	LEU529	0.222	0.015
	ASP569	0.223	0.068
	TYR495	0.223	0.052
	TYR583	0.229	0.035
	ILE531	0.233	0.017
	LYS549	0.235	0.057
	PHE548	0.236	0.023
	LE516	0.238	0.021
	ILE499	0.247	0.028
	THR514	0.251	0.035
	ARG497	0.251	0.034
	THR467	0.256	0.046
	PRO466	0.259	0.067
	GLY528	0.279	0.033

Table S2. Primers used in the present study.

Name	Primer sequence	**Gene ID**
Svx44_F	CGCCCCGTTATTTCATTCTGGTA	
Svx44_R	CGCCGTCAGCCATGATATGC	
Svx45_F	GCATATCATGGCTGACGGCG	
Svx45_R	AGGCGTGTTTTCCTTTTCATTGG	
Svx44His_F	ATCGTCGAGCTCTTAGTGATGGTGATGGTGATGTTTCGCGTAAGCCGGACGTTC	A4U42_RS02180
Svx44His_R	ATCGTCCCATGGCGGATGAAACCTGCGCG	
Svx45His_F	ATCGTCGAGCTCTTAGTGATGGTGATGGTGATGTCTGGCGTAGGCCGGACGTT	A4U42_RS02185
Svx45His_R	ATCGTCCCATGGCGGATGAAACCTGTGTGGCG	
NtEF_F	GCCCAACACTTCTTGATGCTC	LOC107791623
NtEF_R	GACACCAGTTTCCACACGACC	
NtATP_F	GGTCGATGGCTTGGGAGTACC	LOC107762397
NtATP_R	GCACAGATTTACGTTCAATAATACCAG	
NtERF1a_F	GATAGGGCAGCTTATTCAATGAGAGGTC	LOC107766165
NtERF1a_R	CCAAACATTCAAATTCAAGAACTTCCC	
NtACO1_F	CTTGAAGCTGTGCAAGCTGAGGTTAC	LOC107781126
NtACO1_R	CCAAGATTTTCACATAGCAAGTCCAG	
NtLOX_F	CACGCATAATGAGTTTGATAGTTTTG	LOC107806322

NtLOX_R	CTGAGGAGTTGGGTACTTAAATAGGC	
NtAOC_F	CGGCTCAACTGATTCCTCTACAAC	LOC107768393
NtAOC_R	GTTTGTTGCTAAAGGGGACAAGATC	
NtPR1_F	GAGGCGAAAGTCCATACTAATTG	LOC107807832
NtPR1_R	CCACTTGGTCTTTTTATAGATCCAC	
